# Pulmonary blastoma: a case report and review of the literature

**DOI:** 10.1186/1756-0500-7-294

**Published:** 2014-05-13

**Authors:** Robert J Smyth, Aurelie Fabre, Johnathan D Dodd, Waldemar Bartosik, Charles G Gallagher, Edward F McKone

**Affiliations:** 1 St. Vincent’s University Hospital, Elm Park, Dublin 4, Ireland

## Abstract

**Background:**

Pulmonary blastomas are a rare aggressive neoplasm comprising 0.25-0.5% of all primary lung tumors and portend a poor prognosis. They display a biphasic histology with mesenchymal and epithelial components. Historically, the term pulmonary blastoma had included both pure fetal adenocarcinomas, pleuropulmonary blastomas as well as the classic biphasic blastomas. However recent World Health Organisation re-classifications separated well-differentiated fetal adenocarcinomas and pleuropulmonary blastomas from the biphasic tumours.

**Case presentation:**

We present a case of a systemically well 67-year-old Caucasian male who presented with haemoptysis. Investigations confirmed the presence of a large right-sided lung mass and biopsy identified non-small cell carcinoma. The resected tumour was markedly necrotic revealing a biphasic pattern. It was composed of malignant glandular tissue with sub-nuclear vacuoles, associated with a pleomorphic stromal malignant blastematous component, characteristic of classic biphasic pulmonary blastoma.

**Conclusion:**

We present a case of a classic biphasic pulmonary blastoma, a rare lung cancer occurring at an earlier age and portending to poorer prognosis than other more common lung cancers. Given the small number of cases and recent re-classification, interpreting the published epidemiology and clinical features of this disease is difficult. Many earlier reports may have included fetal adenocarcinomas (in particular high grade variant), which need to be considered when discussing treatment and prognosis with newly-diagnosed patients. Much could be gained from a central registry of individual experiences to improve our understanding of this rare lung cancer.

## Background

Pulmonary blastomas are a rare aggressive neoplasm comprising 0.25-0.5% of all primary lung tumors and portend a poor prognosis. Morphologically they mimic fetal lung tissue before 4 months gestation. They display a biphasic histology with mesenchymal and epithelial components. Historically, the term pulmonary blastoma had included both pure fetal adenocarcinomas, pleuropulmonary blastomas as well as the classic biphasic blastomas. However recent World Health Orgnisation (WHO) reclassifications, separated well-differentiated fetal adenocarcinomas and pleuropulmonary blastomas from the classic biphasic tumours. Given it’s rarity and recent re-classification, interpreting the published epidemiology and clinical features of this disease is challenging. We present a case and review the literature of this rare lung cancer.

## Case presentation

A 67-year-old Caucasian man presented to the outpatient department with intermittent frank hemoptysis of 2 months duration. He denied chest discomfort, fever, dyspnea, weight loss or recent travel. He smoked cigarettes for 80 pack years. His past medical history was significant for ischemic heart disease and he was taking aspirin. He was exposed to mycobacterium tuberculosis in the distant past. He worked as a film producer and had no known asbestos exposure. He had recently commenced carpentry classes. On examination he looked well, had no clubbing, but was noted to have mild hyperinflation of the chest with decreased breath sounds. A full blood count, renal and liver chemistries and resting electrocardiograph were all normal. Sputum cytology was also negative.

**Figure 1:**
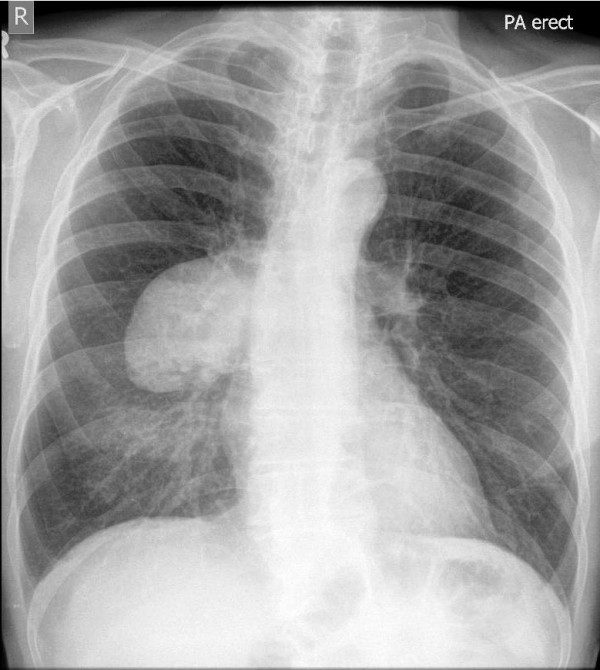
Large right-sided mass with characteristic hilum overlay sign.

**Figure 2:**
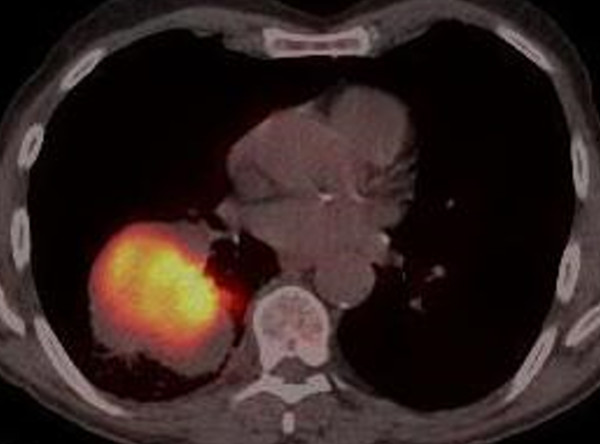
A positron emission tomography-computed tomography (PET-CT) demonstrated increased fludeoxyglucose uptake within the mass, standardized uptake value (SUV max22.1) and no evidence of nodal involvement or distant metastases.

A chest radiograph (Figure 1) revealed a large, well circumscribed mass projected over the right hilum, the so called hilum overlay sign. A subsequent computed tomography (CT) -thorax with intra-venous contrast confirmed a 9.0 × 5.5 cm mass arising from the apical segment of the right lower lobe. It demonstrated homogenous contrast enhancement with no evidence of calcifications or necrosis. The mass abutted the pleura with no sign of invasion. There was no evidence of lymphadenopathy, pleural effusions or osseous metastases. The liver and adrenal glands appeared normal. A positron emission tomography-CT (PET-CT) (Figure 2) demonstrated increased fludeoxyglucose (FDG) uptake within the mass with standardized uptake value (SUV max 22.1) and no evidence of nodal involvement or distant metastases.

**Figure 3:**
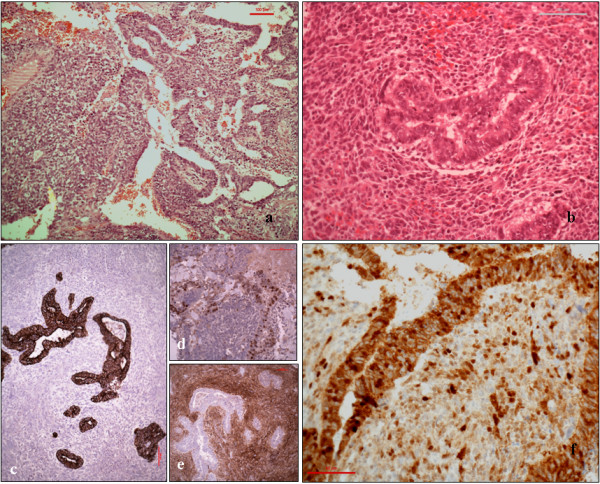
**Histological analysis of the tumour. a.** Malignant glandular tissue with sub-nuclear vacuoles. **b.** Pleomorphic stromal malignant blastematous component. **c.** The malignant glandular component was diffusely positive for the epithelial marker Cam 5.2. **d.** Positive for TTF-1 and negative for vimentin. **e.** The stromal blastematous malignant component showed reverse expression. **f.** The protein beta-cathenin was observed in both components.

A CT-guided biopsy showed a non-small cell primary lung carcinoma with marked necrosis and poor cell differentiation. The subtype could not be more accurately determined. Mediastinoscopy for nodal sampling was negative for nodal metastases. A right pneumenectomy revealed a 9 cm large necrotic mass replacing most of the medial and posterior segments of the right lower lobe. It was a biphasic tumour (Figure 3a) composed of malignant glandular tissue with sub-nuclear vacuoles, associated with a pleomorphic stromal malignant blastematous component (Figure 3b).

The malignant glandular component was diffusely positive for the epithelial marker Cam 5.2 (Figure 3c), and for thyroid transcription factor-1 (TTF-1) (Figure 3d) and negative for vimentin, while the stromal blastematous malignant component showed reverse expression (negative Cam5.2 and TTF-1, positive for vimentin (Figure 3e). The protein beta-cathenin were observed in both components (Figure 3f). Findings were diagnostic of classic biphasic pulmonary blastoma.

## Discussion

Pulmonary blastomas are a rare aggressive neoplasm comprising 0.25-0.5% of all primary lung tumours [1]. Morphologically they mimic fetal lung tissue before 4 months gestation [2].

First described by Barnard in 1952 [3], they have since been divided into three subgroups: classic biphasic pulmonary blastoma, well-differentiated fetal adenocarcinoma -also called monophasic pulmonary blastoma- and pleuropulmonary blastoma of childhood. While well-differentiated fetal adenocarcinoma contains malignant glands and benign appearing mesenchymal tissue and pleuropulmonary blastoma contains malignant glands of embryonal appearance and benign appearing epithelium, classic biphasic pulmonary blastoma contains glands and mesenchymal tissue that are both embryonal and malignant.

In the 1999 and 2004 WHO classifications [4,5], well-differentiated fetal adenocarcinomas and pleuropulmonary blastomas were separated from the biphasic tumours.

Classic biphasic pulmonary blastoma is now considered as part of the spectrum of sarcomatoid carcinomas.

It typically presents with cough, hemoptysis, dyspnea or chest pain due to tumor impinging on the bronchi or pleura. Forty percent of cases may be asymptomatic [6]. The average age at diagnosis is 40 years with an increased frequency in males (2:1) [7]. Clinical examination may reveal localized reduction in breath sounds or sequalae of cigarette smoking with over 80% of cases associated with a smoking history. Abnormalities in laboratory tests are infrequent and non-specific. Pulmonary blastoma almost always presents as a unilateral, large, well-circumscribed, solitary mass on chest radiograph. Given the often, peripheral nature of these tumors, tissue diagnosis by bronchoscopy only occurs in 25% of cases [6] but they can often be visualized on thoracic ultrasound with findings correlating well with those seen on CT [8].

The role of PET-CT in the radiological staging of pulmonary blastoma is unknown with our case demonstrating final pathological staging consistent with that observed using PET-CT. Due to the challenging nature of the histology, a preoperative diagnosis is only obtained in one third of cases [1] Differential diagnoses must include benign conditions such as hamartoma and pleural fibroma as well as malignant conditions such as other primary or metastatic lung cancers.

Pulmonary blastomas are biphasic tumours that are part of the sarcomatoid carcinoma subgroup [4], which also include carcinosarcomas (defined as a malignant tumour having a mixture of carcinoma- and sarcoma-containing heterologous elements such as malignant cartilage, bone, or skeletal muscle) and pleomorphic carcinomas (similar tumour without heterologous elements), both histologically resembling adult-type carcinomas and sarcomas.

The epithelial component of classic biphasic blastoma is composed of tubules of glycogen-rich, non-ciliated cells that resemble fetal lung of pseudo-glandular stage of lung development with sub-nuclear and supra-nuclear glycogen vacuoles. The embryonic appearance of the stroma is due to the small size, oval and spindle shape of the cells, and myxoid matrix. Classically this blastematous stroma does not express cytokeratins or pulmonary markers.

Beta-catenin may play a role in tumorigenesis of classic pulmonary blastoma. Its aberrant nuclear/cytoplasmic localization by immunostaining has been reported to be useful in distinguishing classic pulmonary blastoma from a blastomatoid variant of carcinosarcoma and from high-grade fetal type adenocarcinomas [9].

Surgery is the optimal treatment for localized disease. A mean survival of 33 months was reported in resected cases compared to 2 months in those with un-resected disease. Limited resections do better than pneumonectomies [7], presumably due to less extensive tumor burden. Larsen [7] reported a 16% response rate in to chemotherapy in 43 cases of classic biphasic pulmonary blastoma. No agent is known to be more effective than another, but cisplatin is often used given its efficacy with primitive tumors. Most cases have not shown a response to radiotherapy.

Prognosis is poor with two thirds of patients dying within 2 years and only a 16% 5-year survival. Prognosis is determined by size of the tumor at time of diagnosis, with tumors <5 cm doing better. Tumor metastasis and tumor recurrence despite resection both predict a poor prognosis. Unfortunately, 43% of tumors recur within 1 year with a propensity for sites such as brain and mediastinum [6]. Recurrence tends to occur within 1 year after diagnosis or not at all [7].

## Conclusions

We present a case of a classic biphasic pulmonary blastoma, a rare lung cancer occurring at an earlier age and portending to poorer prognosis than other more common lung cancers. Recurrence after resection is high and regular surveillance is recommended especially within the first year. Our patient opted not to undergo adjuvant chemotherapy and remains disease free at 23 months.

Given the small number of cases and recent re-classification, interpreting the published epidemiology and clinical features of this disease is difficult. Many earlier reports may have included fetal adenocarcinomas (in particular high grade variant), which needs to be considered when discussing treatment and prognosis with newly diagnosed patients. Much could be gained from a central registry of individual experiences to improve our understanding of this rare lung cancer.

## Consent

Written informed consent was obtained from the patient for publication of this Case Report and any accompanying images. A copy of the written consent is available for review by the Editor-in-Chief of this journal.

## Competing interests

The authors declare that they have no competing interests.

## Authors’ contributions

RS researched previous publications on the topic with written contribution, AF contributed to pathology discussion, JD to radiology aspects, WB on surgical treatments, CG and EMcK on clinical and therapeutics. All authors read and approved the final manuscript.
